# Supplementary material for: Zeb1 promotes corneal neovascularization by regulation of vascular endothelial cell proliferation
Source: Commun Biol. 2020 Jul 3;3:349. doi: 10.1038/s42003-020-1069-z (PMC7335040; doi:10.1038/s42003-020-1069-z)
Supplement: Supplementary file 1 — Supplementary Information [file 42003_2020_1069_MOESM1_ESM.pdf]

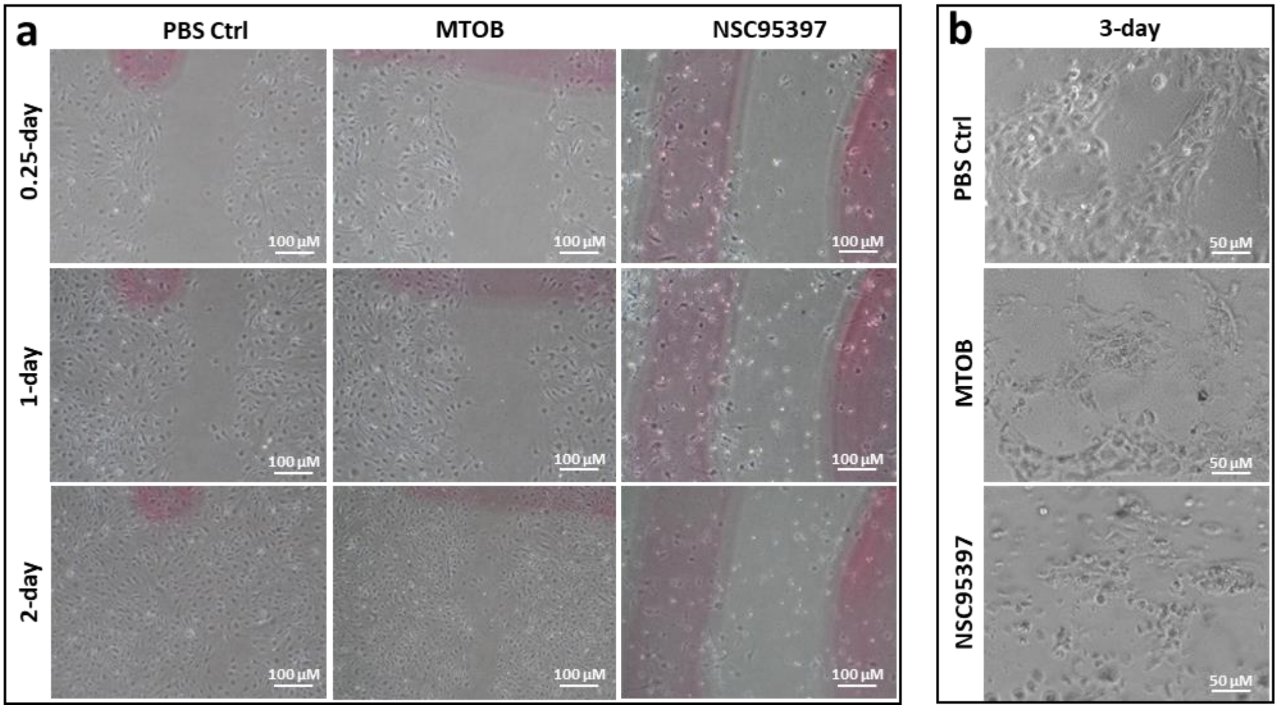

Supplementary Figure 1. Treatment with the ZEB1-CtBP inhibitors causes reduction of mRMVEC migration and tube formation. Representative images to show that compared to the PBS control, both 10 mM MTOB and 10 µM NSC95397 treatments reduced (**a**) mRMVEC migration and (**b**) tube formation.

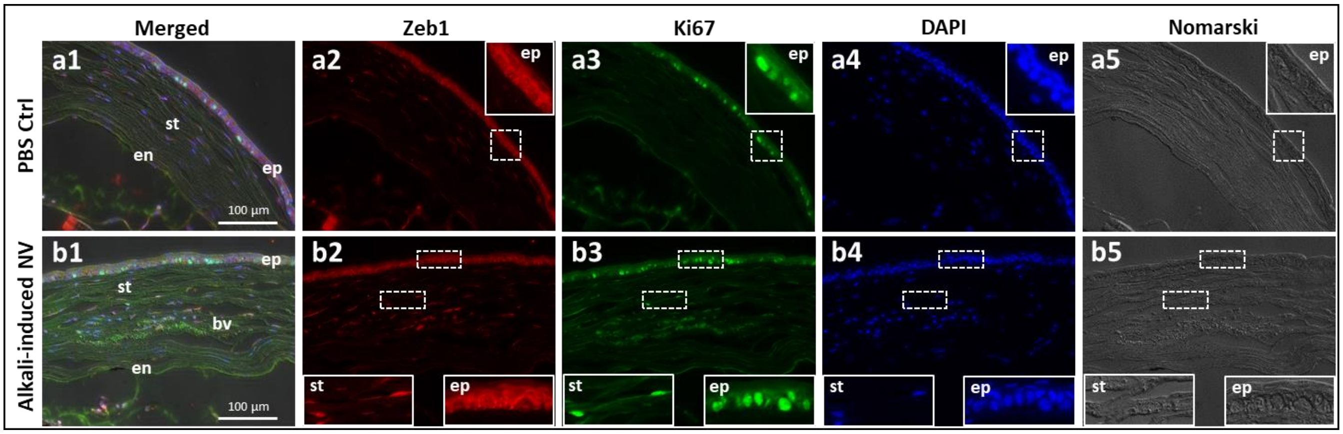

Supplementary Figure 2. Effects of the alkali treatment on corneal epithelial and stromal cell proliferation, inflammation, and NV. (a) Compared to the PBS control, **(b)** the alkali treatment induces cell proliferation in both the epithelium and stroma of the cornea, and inflammation and NV in the stroma**. ep,** epithelium; **st,** stroma; **bv,** blood vessel; **en,** endothelium.

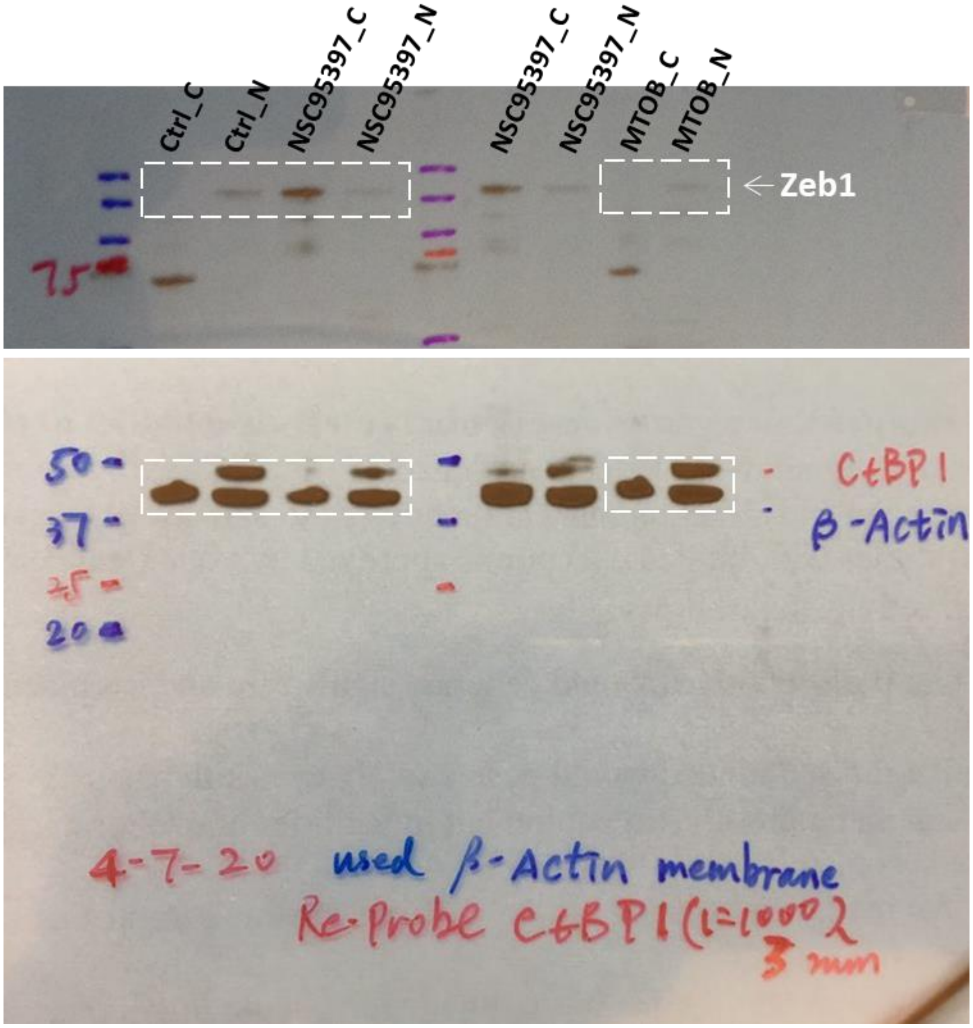

Supplementary Figure 3. Raw blots for Fig. 5c.

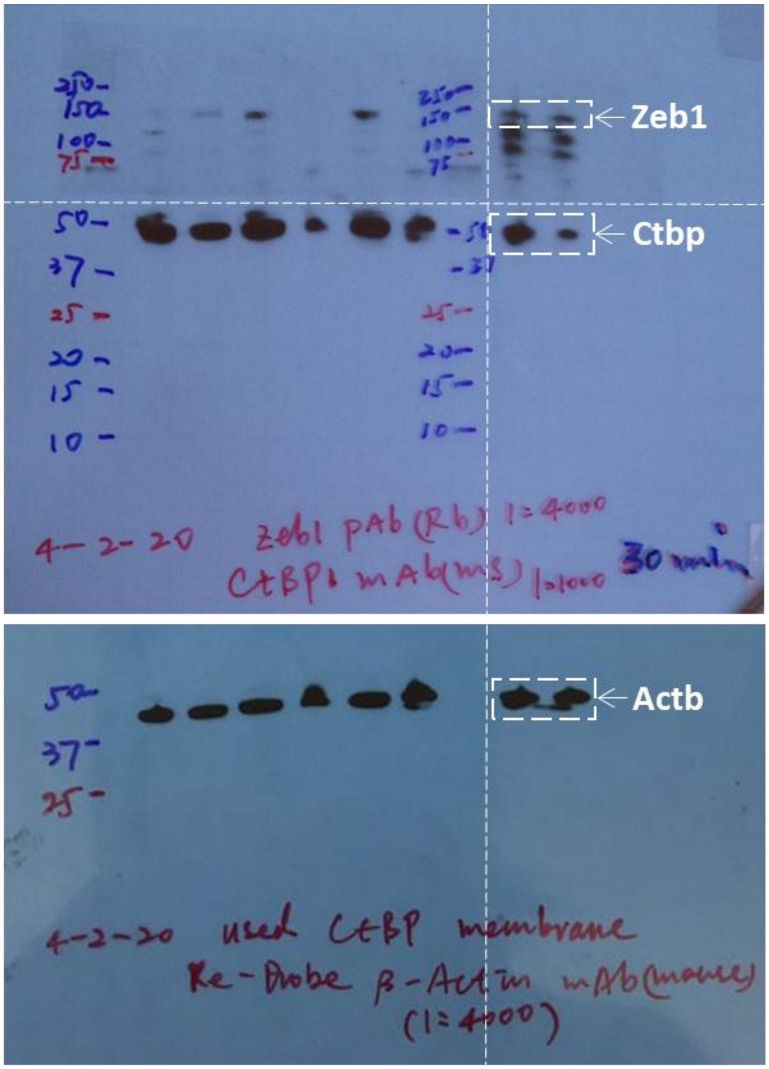

Supplementary Figure 3. Raw blots for Fig. 6a.

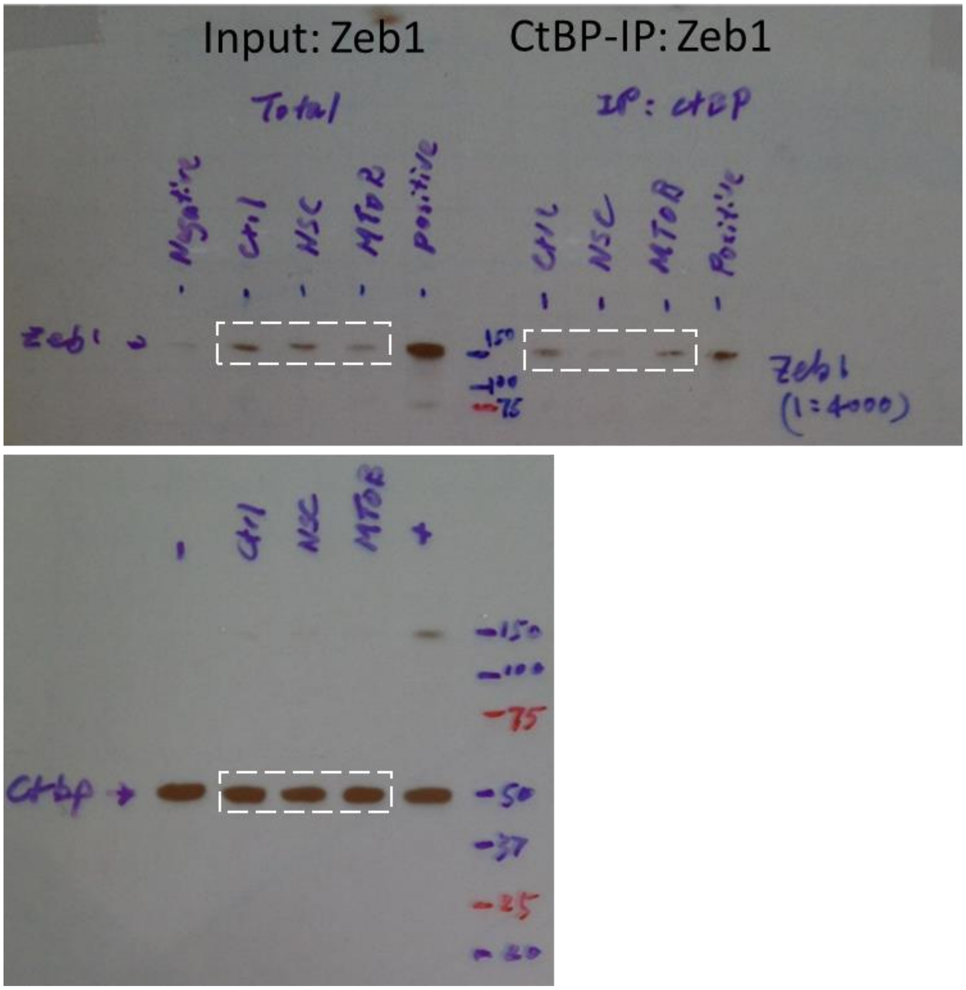

Supplementary Figure 3. Raw blots for Fig. 6e.

Supplementary Table 1. Primers used for qPCR detection

Primer name	Sequence (5'—3')	Tm ˚C	Amplicon (bp)
Mm Zeb1 LP	TGGCAAGACAACGTGAAAGA	60.0	200
Mm Zeb1 RP	AACTGGGAAAATGCATCTGG	60.0	
Mm Vegfa LP	GCTTCCTACAGCACAGCAGATG	58.1	158
Mm Vegfa RP	CCGCTCTGAACAAGGCTCACA	59.8	
Mm Vegfb LP	CCTGACGATGGCCTGGAATGT	59.6	163
Mm Vegfb RP	TGTCTGGCTTCACAGCACTCTC	59.2	
Mm Vegfc LP	GCTTCTTGTCTCTGGCGTGTTC	58.4	168
Mm Vegfc RP	GCAACTGCTCCTCCAGGTCTT	59.5	
Mm Vegfr1 LP	TTAGGGGGTTCTCCATACCC	55.7	221
Mm Vegfr1 RP	TCTTTCCCATCCTGTTGGAC	54.5	
Mm Vegfr2 LP	TCTTTCGGTGTGTTGCTCTG	55.3	214
Mm Vegfr2 RP	CCAAATGCTCCACCAACTCT	55.3	
Mm Vegfr3 LP	GCCCTACTGCAAGGTGCTAC	58.2	196
Mm Vegfr3 RP	CCACATCGAGCTCTTCCTGT	56.6	
Mm p14 LP	TGAGGCTAGAGAGGATCTTGAGA	56.3	91
Mm p14 RP	GCAGAAGAGCTGCTACGTGAA	57.3	
Mm p15 LP	CCCTGCCACCCTTACCAGA	59.3	169
Mm p15 RP	CAGATACCTCGCAATGTCACG	55.7	
Mm p16 LP	CCCAACGCCCCGAACT	54.3	79
Mm p16 RP	GCAGAAGAGCTGCTACGTGAA	61	
Mm p21 LP	GTGGCCTTGTCGCTGTCTT	58.1	126
Mm p21 RP	GCGCTTGGAGTGATAGAAATCTG	55.9	
Mm p27 LP	TTGGGTCTCAGGCAAACTCT	56.3	157
Mm p27 RP	TCTGTTCTGTTGGCCCTTTT	54.7	
Mm p57 LP	GGAGCAGGACGAGAATCAAG	55.3	189
Mm p57 RP	ACGTTTGGAGAGGGACACC	57.1	
Mm IL-1b LP	ACTCATTGTGGCTGTGGAGA	56.3	199
Mm IL-1b RP	TTGTTCATCTCGGAGCCTGT	56.2	
Mm TNF LP	CGTCGTAGCAAACCACCAAG	56.4	241
Mm TNF RP	GGCAGAGAGGAGGTTGACTT	56.6	
Mm GAPDH LP	AACGACCCCTTCATTGAC	56.0	191
Mm GAPDH RP	TCCACGACATACTCAGCAC	56.0	
miR200c LP	cgtcttacccagcagtgtttgg	58.2	98
miR200b LP	catcttactgggcagcattgga	57.4	98
miR200a LP	catcttaccggacagtgctgga	58.8	98
