# Supplementary material for: Zeb1 promotes corneal neovascularization by regulation of vascular endothelial cell proliferation
Source: Commun Biol. 2020 Jul 3;3:349. doi: 10.1038/s42003-020-1069-z (PMC7335040; doi:10.1038/s42003-020-1069-z)
Supplement: Supplementary file 2 — Description of Additional Supplementary Files [file 42003_2020_1069_MOESM2_ESM.pdf]

Description of Additional Supplementary Files

Supplementary data 1: Fig. 1d data - Zeb1 genotypes and their lung tissue phenotypic measurements

Supplementary data 2: Fig. 2b data - Scores of 1N NaOH-induced NV on wt and het Zeb1 mouse corneas

Supplementary data 3: Fig. 3c-d data - Number of Cd31+/Zeb1+ vascular endothelial cells and number of vessels counted based on 4 cryosections of 2 PBS control and 2 alkali-treated corneas

Supplementary data 4: Fig. 4e data - Effect of Zeb1 knockdown by shRNA on mRMVEC proliferation

Supplementary data 5: Fig. 5b data - Percentages of mRMVEC with cytoplasmic Zeb1 after treated with NSC95397 or MTOB for 1 day; Fig. 5e data - Cell proliferation rates of mRMVEC treated with MTOB or NSC95397; Fig. 5f data - mMRVEC cell migration assays; Fig. 5g data - Tube formation of mRMVEC cells treated with chemicals

Supplementary data 6: Fig. 6d data - Proliferation assay on mRMVEC knocked down by Ctbp shRNA; Fig. 6g-i data - Scores of alkali-induced NV after treated with 2 drugs
